# L-theanine attenuates AlCl3-induced cognitive dysfunction and neurotoxicity in rats: a behavioral and biochemical study

**DOI:** 10.55730/1300-0144.6375

**Published:** 2026-04-13

**Authors:** Muhammet Oğuzhan DÖNMEZ, Azize ŞENER, Şeyda KARAMAN ERSOY, Mahdi MARZİ, Göksel ŞENER

**Affiliations:** 1 Division of Pharmaceutical Sciences, Department of Pharmacology, Faculty of Pharmacy, Fenerbahçe University, İstanbul, Turkiye; 2 Division of Basic Pharmaceutical Sciences, Department of Biochemistry, Faculty of Pharmacy, Fenerbahçe University, İstanbul, Turkiye; 3 Division of Basic Pharmaceutical Sciences, Department of Analytical Chemistry, Faculty of Pharmacy, Fenerbahçe University, İstanbul, Turkiye; 4 Division of Basic Pharmaceutical Sciences, Department of Pharmaceutical Microbiology, Faculty of Pharmacy, Fenerbahçe University, İstanbul, Turkiye

**Keywords:** Aluminum chloride, Alzheimer’s disease, L-theanine, cognitive dysfunction, oxidative stress

## Abstract

**Background/aim:**

The effects of L-theanine (LTN), a natural compound found in tea leaves, on biochemical parameters and cognitive functions were investigated in an aluminum chloride (AlCl_3_)-induced Alzheimer’s disease-like experimental model.

**Materials and methods:**

A total of 32 male Wistar rats were allocated into four groups: control, AlCl_3_-induced AD-like group, LTN-treated AD-like group, and donepezil-treated AD-like group. Donepezil was used as a reference drug. For the induction of AlCl_3_-induced neurotoxicity, AlCl_3_ was administered by gavage at a dose of 25 mg/kg/day during the 1st week and 150 mg/kg/day for the subsequent 3 weeks. LTN was administered orally at a dose of 100 mg/kg. Spatial memory was assessed using the Morris water maze and the novel object recognition test. Brain tissues were collected to analyze acetylcholinesterase, catalase, and myeloperoxidase activities, as well as amyloid beta (Aβ), lipid peroxidation, glutathione, and nitric oxide levels.

**Results:**

LTN reversed AlCl_3_-induced cognitive deficits. Moreover, the elevations in acetylcholinesterase and myeloperoxidase activities, as well as increased Aβ levels in the brain tissues of AlCl_3_-treated rats, were significantly reduced following LTN treatment. The increases in lipid peroxidation and nitric oxide observed in the AlCl_3_ group were suppressed by LTN treatment, while decreased antioxidant defenses, namely reduced glutathione levels and catalase activity, were restored.

**Conclusion:**

The findings demonstrate that AlCl_3_ exposure impairs cholinergic function, induces oxidative damage, and leads to cognitive dysfunction, whereas LTN treatment effectively mitigates these effects, suggesting potential neuroprotective benefits against aluminum-induced neurotoxicity.

## 1. Introduction

Alzheimer’s disease (AD) remains the predominant cause of dementia. The pathological hallmarks of the disease encompass amyloid beta (Aβ) accumulation, neurofibrillary tangle formation, progressive synaptic and neuronal loss, and elevated acetylcholinesterase activity. Alpha-secretase-mediated cleavage of amyloid precursor protein produces soluble fragments, while cleavage by beta- and gamma-secretases leads to the increased formation of insoluble and neurotoxic Aβ peptides, which accumulate in the cerebral parenchyma and manifest as senile plaques [1]. Although the cholinergic and amyloid hypotheses are the two main theories regarding the etiology of AD, the disease is also influenced by a number of risk factors, including aging, head trauma, vascular disorders, genetics, infections, and environmental variables. Furthermore, these factors are known to contribute to oxidative stress, which is associated with neuronal death and the buildup of Aβ [2].

In the brain, acetylcholine (ACh) is involved in several physiological functions including learning, memory, attention, and sensory information processing [3]. De Ferrari et al. [4] showed that acetylcholinesterase **(**AChE), the enzyme responsible for the hydrolysis of ACh, interacts with Aβ peptides and increases amyloid fibril formation, supporting a role of enhanced enzyme activity in the pathogenesis of AD [5]. In Alzheimer’s disease, Aβ accumulation is thought to contribute to cholinergic neuronal degeneration, reduce neuronal choline uptake, and consequently diminish ACh release, thereby disrupting cholinergic neurotransmission and leading to cognitive decline and memory loss. Therefore, disruption of the cholinergic system, characterized by decreased ACh levels in the central nervous system and/or overactivity of AChE, has been considered a major contributor to the cognitive impairment observed in AD [6].

Aluminum is a potential neurotoxic agent that is abundant in the environment and can cause oxidative damage to various cellular components. Aluminum enters the body through drinking water, food, drugs, vaccines, and aluminum-containing kitchenware. Chronic exposure to aluminum, which accumulates in the brain, has been shown to induce oxidative stress and neuronal damage, ultimately contributing to AD, a condition known to be associated with cholinergic dysfunction [7–9].

L-theanine (LTN) occurs naturally in green tea (*Camellia sinensis*), with tea leaves representing its principal source. Although it is also present in black and white tea, green tea contains the highest concentration. Chemically, it is an analog of L-glutamic acid and is a water-soluble compound [10]. LTN is thought to have protective properties against neurodegenerative conditions such as Parkinson’s disease and Aβ-related AD pathology [11–13]. It also reduces oxidative stress and free radical formation, thereby preventing cellular damage. By influencing the levels of gamma-aminobutyric acid (GABA), dopamine, and serotonin in the brain, LTN promotes relaxation and stress reduction while maintaining alertness and cognitive function. Furthermore, studies suggest that LTN enhances attention, strengthens memory, and improves cognitive performance [14].

AD is a progressive neurodegenerative disease marked by neuronal injury and memory impairment. The present study aimed to elucidate the mechanisms by which AlCl_3_ induces brain injury, as well as to evaluate the protective effects of LTN against this damage and the associated cognitive deficits.

## 2. Materials and methods

### 2.1. Animals

Male Wistar albino rats aged 10 weeks and weighing 200–300 g were used. Ethical approval for the study was obtained from the İstanbul Medeniyet University Animal Experiments Ethics Committee (protocol no: 2025/2-1). Housing conditions were standardized to a temperature of 22 °C ± 2 °C and a relative humidity of 50% ± 5%. Lighting was controlled on a 12 h day and 12 h night cycle.

### 2.2. Drugs and chemicals

The rat Aβ peptide 1–42 (Aβ1–42) ELISA kit was obtained from Sinogeneclon Co Ltd. (cat. no: SG-20347; Hangzhou, China). Aluminum chloride (AlCl_3_) (cat. no: 294713) and 5,5′-dithiobis-(2-nitrobenzoic acid) (DTNB) (cat no: D8130) were purchased from Sigma-Aldrich (St. Louis, MO, USA), and donepezil hydrochloride monohydrate (cat. no: D6821) was obtained from Scientific Laboratory Supplies Ltd. (Nottingham, UK). L-theanine was kindly supplied by Orzax Pharmaceuticals Inc. (İstanbul, Türkiye) as a commercial dietary supplement. Other reagents used in the study were of analytical grade and supplied by Merck KGaA (Darmstadt, Germany). AlCl_3_ was freshly dissolved in distilled water before administration. L-Theanine was prepared in distilled water immediately before use. Donepezil hydrochloride monohydrate was freshly dissolved in normal saline (0.9% NaCl) prior to intraperitoneal administration.

### 2.3. Experimental design

A total of 32 animals were separated into four groups (n = 8 per group) as follows:

Control group (C): Received oral saline throughout the experimental period.

AlCl_3_-induced AD-like group (AD): The model was induced by oral administration of AlCl_3_ with a gradual dose escalation protocol, 25 mg/kg/day for the 1st week and 150 mg/kg/day for the following 3 weeks [15]. The gradual increase in dose was employed to minimize acute systemic toxicity and animal mortality while enabling sustained aluminum exposure sufficient to induce Alzheimer-like cognitive impairment and neurodegenerative alterations, as described in experimental models. Although this dosing protocol may appear relatively high, it was selected based on previously established experimental models using AlCl_3_ to induce Alzheimer-like neurotoxicity in rats [16,17].

LTN-treated AD-like group (AD + LTN): Received AlCl_3_ as in the AD-like group and was treated orally with LTN (100 mg/kg/day) for the same period. The LTN dose (100 mg/kg, p.o.) was selected based on previous experimental animal studies demonstrating neuroprotective and antioxidant efficacy at this level, along with a favorable tolerability profile [18–20].

DNP-treated AD-like group (AD + DNP): Received AlCl_3_ as in the AD-like group and was treated with DNP (3 mg/kg/day, intraperitoneally). The DNP dose was chosen according to Kadıoğlu-Yaman et al. [21]. DNP was administered via the intraperitoneal route to enhance the reliability of its central effects by minimizing variability associated with oral bioavailability, particularly in view of the potential gastrointestinal effects of high-dose oral AlCl_3_ administration.

The novel object recognition test (NORT) and Morris water maze (MWM) test were used to evaluate cognitive performance by the end of the experiment.

### 2.4. Behavioral assessments

#### 2.4.1. Novel object recognition test

The test consists of two phases. Rats were initially habituated to the open-field apparatus (65 × 45 × 65 cm) for 10 min. Familiarization was conducted within the same arena by presenting two identical objects (F + F) for an additional 10 min. Subsequently, the animals were left undisturbed in their respective home cages for 1 h. During the test session, each rat was reintroduced to the same arena, which contained one familiar object (F) and one novel object (N). The novel object differed in both color and shape from the familiar object. Object exploration was operationally defined as the rat directing its nose toward an object at a distance of ≤2 cm. The rats’ exploration time was recorded for 3 min. To avoid residual odor cues, the box and objects were thoroughly wiped with 70% ethanol before subsequent testing [22]. The exploration times for the familiar and novel objects, as well as the preference index (PI) and discrimination index (DI), were calculated using equations reported in previous studies [23]. The time spent on the familiar object was defined as time on the familiar object (TF), and the time spent on the novel object was defined as time on the novel object (TN).

Discrimination index: (TN − TF) / (TN + TF)

Preference index: TN / (TN + TF)

#### 2.4.2. Morris water maze test

The memory and learning abilities of rats were evaluated using the MWM test in a circular pool with a diameter of 1.2 m and a height of 0.47 m. The pool contained black water (24–25 °C; 20 cm deep). Colored cardboards were placed on the walls around the pool so that the rats could recognize their surroundings. The pool was divided into four equal quadrants, and two primary axes were determined [24].

The MWM test consists of a 4 day training period followed by a test trial. During the training period, a platform, referred to as the escape platform, with a diameter of 10 cm and the same color as the water, was placed in the target quadrant, centered and submerged 2 cm below the water surface [23]. Each rat was randomly placed in the pool from one of the quadrants and allowed to swim for up to 75 s to find the platform, while video recording was performed. If the platform was not reached within 75 s, the animal was guided to it, and a 75-s latency was recorded. Then, the animals were kept in their cages for 60 s before being returned to the pool for platform-finding trials from each quadrant.

On the 5th day, a probe trial was conducted by removing the concealed platform and permitting the rats to swim freely for 60 s. Time in the target quadrant was measured.

### 2.5. Tissue collection and homogenization

The animals were decapitated under deep anesthesia (ketamine 90 mg/kg + xylazine 10 mg/kg, i.p.). After decapitation, brain tissues were rapidly removed, rinsed with ice-cold physiological saline to remove excess blood, and stored at −80 °C until biochemical analyses. For biochemical measurements, tissues were homogenized under cold conditions in physiological saline to obtain 10% (w/v) homogenates. No protease inhibitors were used during homogenization.

### 2.6. Biochemical analysis

#### 2.6.1. Analysis of AChE activity

AChE activity in brain tissue was measured using a kinetic photometric method [25]. In this assay, acetylcholinesterase hydrolyzes acetylcholine into thiocholine and acetate. The concentration of thiocholine was determined by measuring the absorbance of the yellow-colored 5-thio-2-nitrobenzoic acid (TNB) formed during its reaction with DTNB. The intensity of the yellow color, which correlates with AChE activity, was measured spectrophotometrically at 412 nm, and the results were expressed as μmol/min/mg protein.

#### 2.6.2. Analysis of brain Aβ levels

Brain Aβ levels were analyzed using an ELISA kit. The analysis was performed according to the manufacturer’s instructions.

#### 2.6.3. Analysis of lipid peroxidation, myeloperoxidase activity, and nitric oxide levels

To assess lipid peroxidation in brain tissue, the amount of malondialdehyde (MDA), which forms a pink chromogen upon reaction with thiobarbituric acid (TBA), was measured spectrophotometrically as previously described by Buege and Aust [26]. The results were expressed as nmol/g tissue.

Myeloperoxidase (MPO) activity in brain tissue was measured according to the method described by Hillegass et al. [27]. Brain tissue was homogenized in 50 mM potassium phosphate buffer (pH 6.0) and centrifuged. The pellet was then resuspended in 50 mM phosphate buffer containing 0.5% hexadecyltrimethylammonium bromide (HETAB). Then, it was freeze–thawed three times. A total of 1.15 mL of a reaction mixture comprising 20 mM H_2_O_2_, o-dianisidine, and 50 mM phosphate buffer was mixed with the samples. Absorbance readings were obtained at 460 nm. MPO activity (U) was defined as the amount of enzyme that converts 1 μmol of H_2_O_2_ per minute. The results were expressed as U/mg protein.

NO levels were determined by measuring its metabolites, nitrite and nitrate [28]. Nitrate was reduced to nitrite by vanadium (III) chloride. In the presence of sulfanilamide, N-(1-naphthyl)ethylenediamine dihydrochloride reacted with nitrite to form a diazonium compound. Absorbance of the resulting chromophore was read at 540 nm using a spectrophotometer. Data were reported as μmol/mg protein.

#### 2.6.4. Analysis of reduced glutathione levels and catalase activity

The levels of reduced glutathione (GSH) in brain tissue were quantified using the method described by Beutler’s [29]. The sulfhydryl groups in the deproteinized samples reacted with Ellman’s reagent (DTNB) to form a colored product. The concentration of the product was determined spectrophotometrically. The brain GSH levels were expressed as μmol/g tissue.

Brain CAT activity was measured according to the method described previously [30]. The results were expressed as U/mg protein.

#### 2.6.5. Analysis of protein levels

Protein content of the tissue samples was determined using the method described by Bradford [31], with bovine serum albumin as the standard.

### 2.7. Statistical analysis

All statistical analyses were performed using GraphPad Prism 9 (GraphPad Software, San Diego, CA, USA). Data were expressed as mean ± SEM. Biochemical data were analyzed by one-way ANOVA followed by Tukey’s post hoc test, and behavioral data by two-way ANOVA.

## 3. Results

### 3.1. Effects of LTN on behavioral alterations in AlCl_3_-induced rats

**Figure 1:**
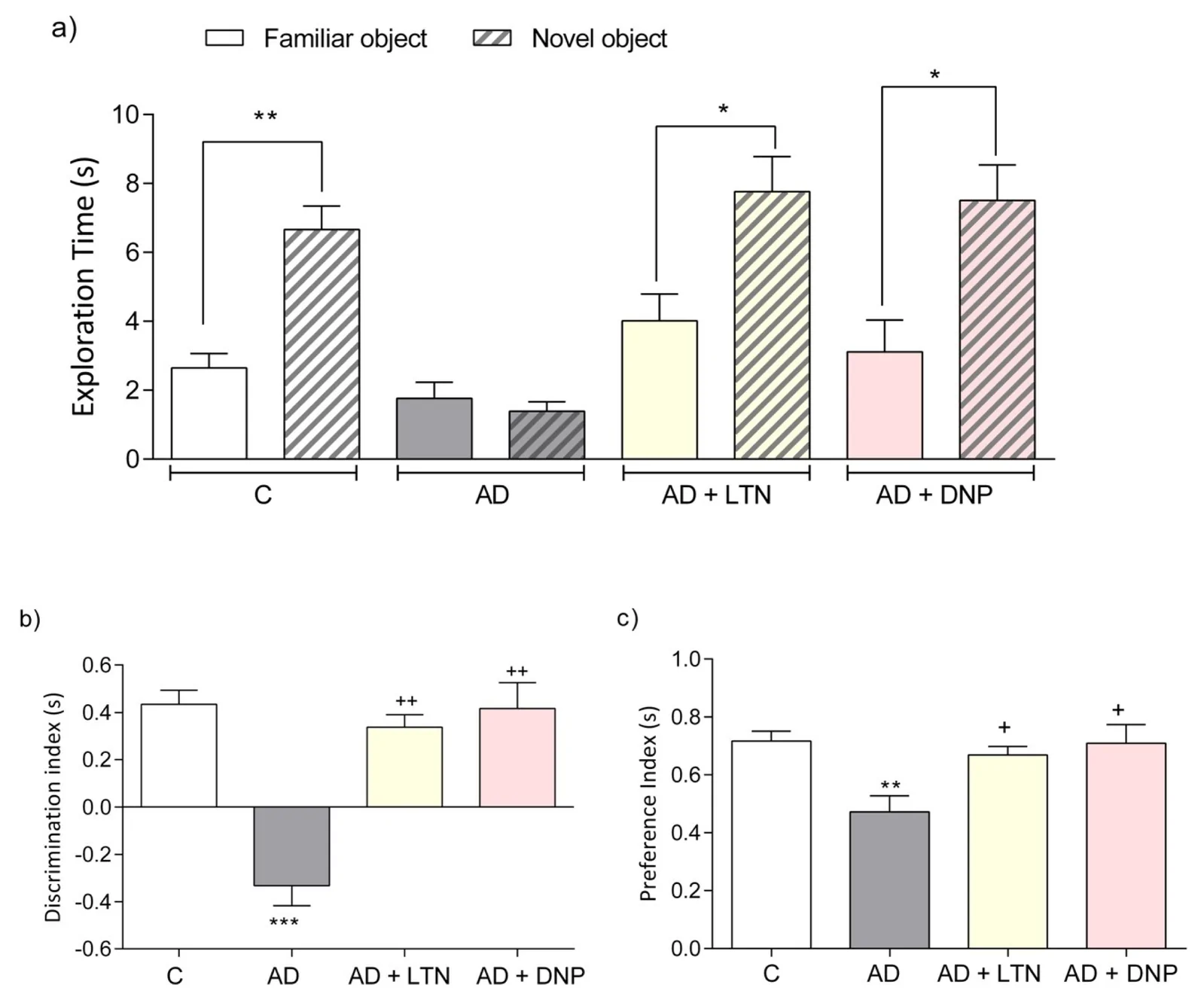
Novel object recognition test (NORT): (a) Exploration time (*p < 0.05, **p < 0.01 versus familiar object). (b) Discrimination index (DI) (***p < 0.001 versus control group; ++p < 0.01 versus AD group). (c) Preference index (PI) (**p < 0.01 versus control group; +p < 0.05 versus AD group). LTN: L-theanine; DNP: donepezil; AD: Alzheimer’s disease.

Learning and memory in rats were assessed using the NORT. In the AD group, exploration capacity was decreased in the novel object recognition test. The exploration time of the novel object and the recognition index were increased in the LTN- and DNP-treated groups compared with the AD group (Figure 1a). When the discrimination and preference index values calculated from the exploration times were examined, the DI and PI values were significantly lower in the AD group, whereas both values in the LTN-treated group were close to the control group and significantly increased compared with the AD group, similar to the DNP-treated group (Figures 1b and 1c).

**Figure 2:**
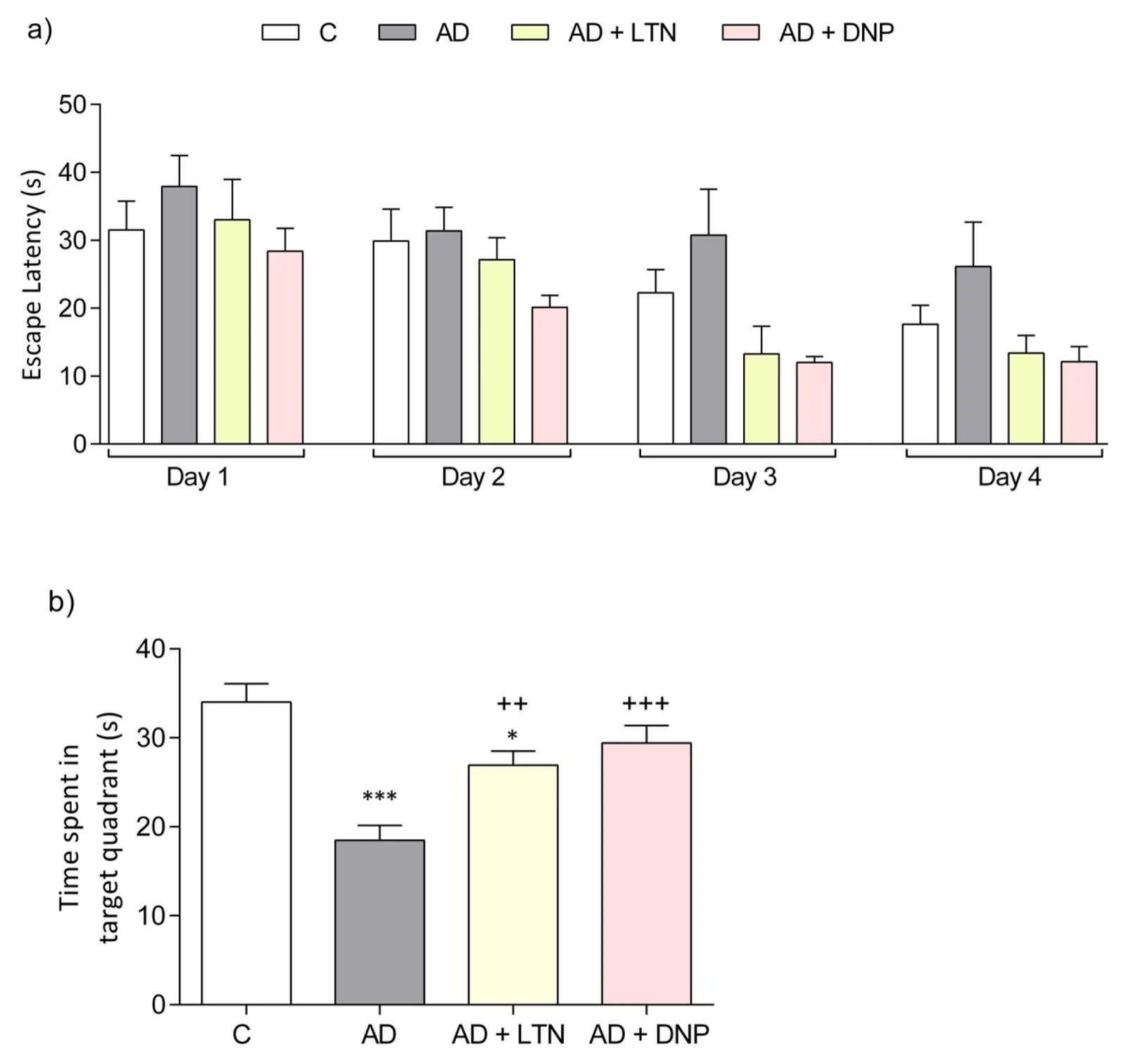
(a) Performances in the Morris water maze (MWM) training period. +p < 0.05, ++p < 0.01 versus AD group for each training day. (b) Probe test performance in the MWM. *p < 0.05, ***p < 0.001 versus control group; ++p < 0.01, +++p < 0.001 versus AD group. LTN: L-theanine; DNP: donepezil; AD: Alzheimer’s disease.

In the MWM training trials, the performances of the groups on day 1 did not differ significantly (Figure 2a). Post hoc comparisons for each day revealed significant differences among the groups on training days 3 and 4. Data collected during the 4-day MWM training period were analyzed using a two-way ANOVA (factors: treatment group × training day). The analysis revealed significant effects of treatment group (F(3,112) = 15.34; p < 0.0001) and training day (F(3,112) = 9.89; p < 0.0001), with no significant interaction between the factors (F(9,112) = 0.66; p = 0.74).

During the MWM probe session, the percentage of time the rats spent in the target quadrant reflected their memory performance (Figure 2b). In the probe test, the AD group showed significantly lower performance compared with the control group (p < 0.001). On the other hand, the LTN- and DNP-treated AD groups (p < 0.01–0.001) displayed higher memory performance than the saline-treated AD group.

### 3.2. Effects of LTN on biochemical alterations in AlCl_3_-induced rats

**Figure 3:**
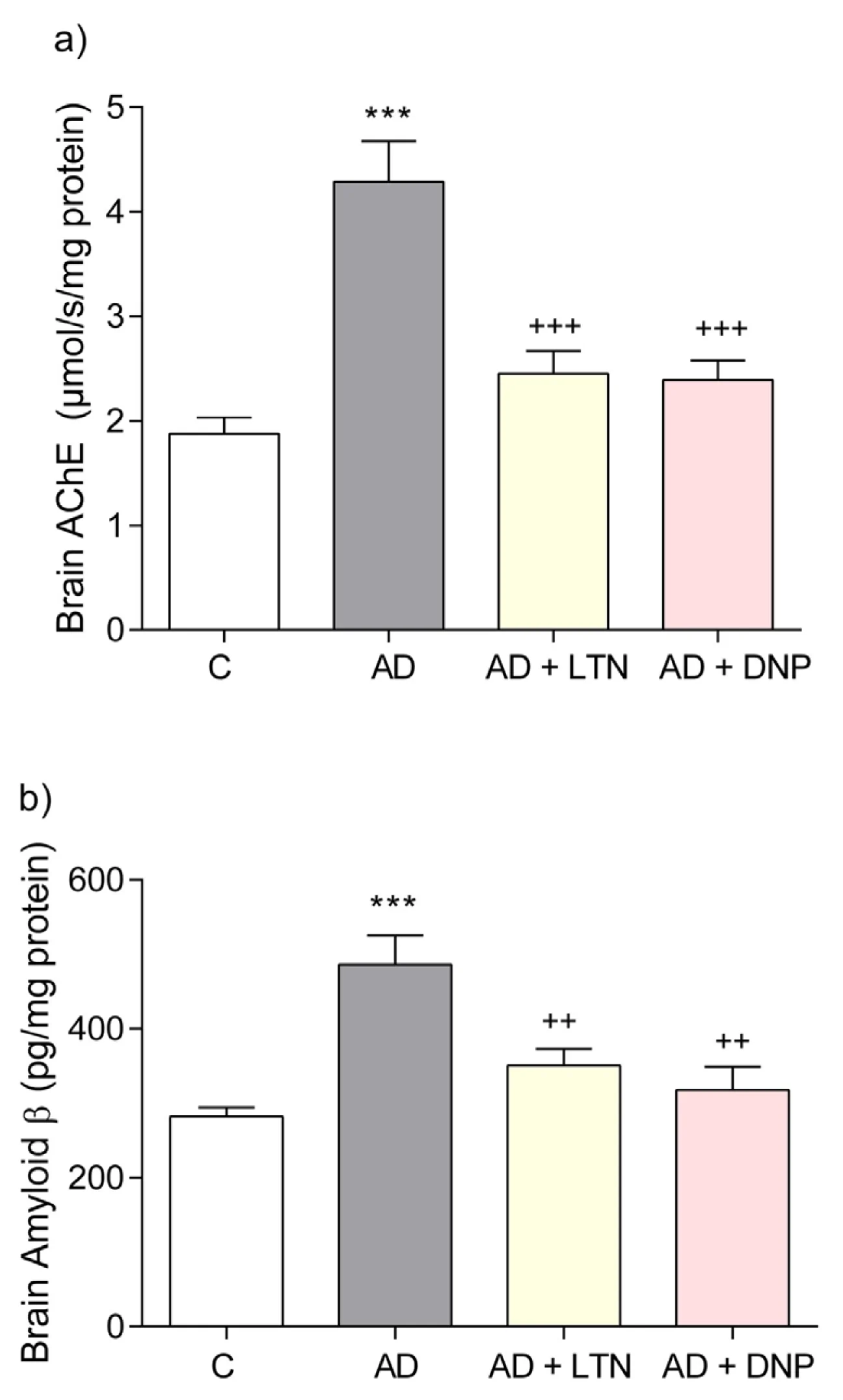
Effects of LTN on AlCl_3-_induced acetylcholinesterase (AChE) activity (a) and amyloid β levels (Aβ) (b) in brain tissue. ***p < 0.001 versus control group; ++ p < 0.01, +++ p < 0.001 versus AD group. C: control; AD: Alzheimer’s disease; LTN: L-theanine; DNP: donepezil.

In the AlCl_3_-induced AD-like group, brain Aβ levels and AChE activity were significantly increased (by 74% and 148%, respectively; p < 0.01; Figure 3a). However, treatment with either LTN (by 28% and 43%, respectively) or DNP (by 35% and 47%, respectively) significantly reduced these parameters (p < 0.01; Figure 3b).

**Figure 4:**
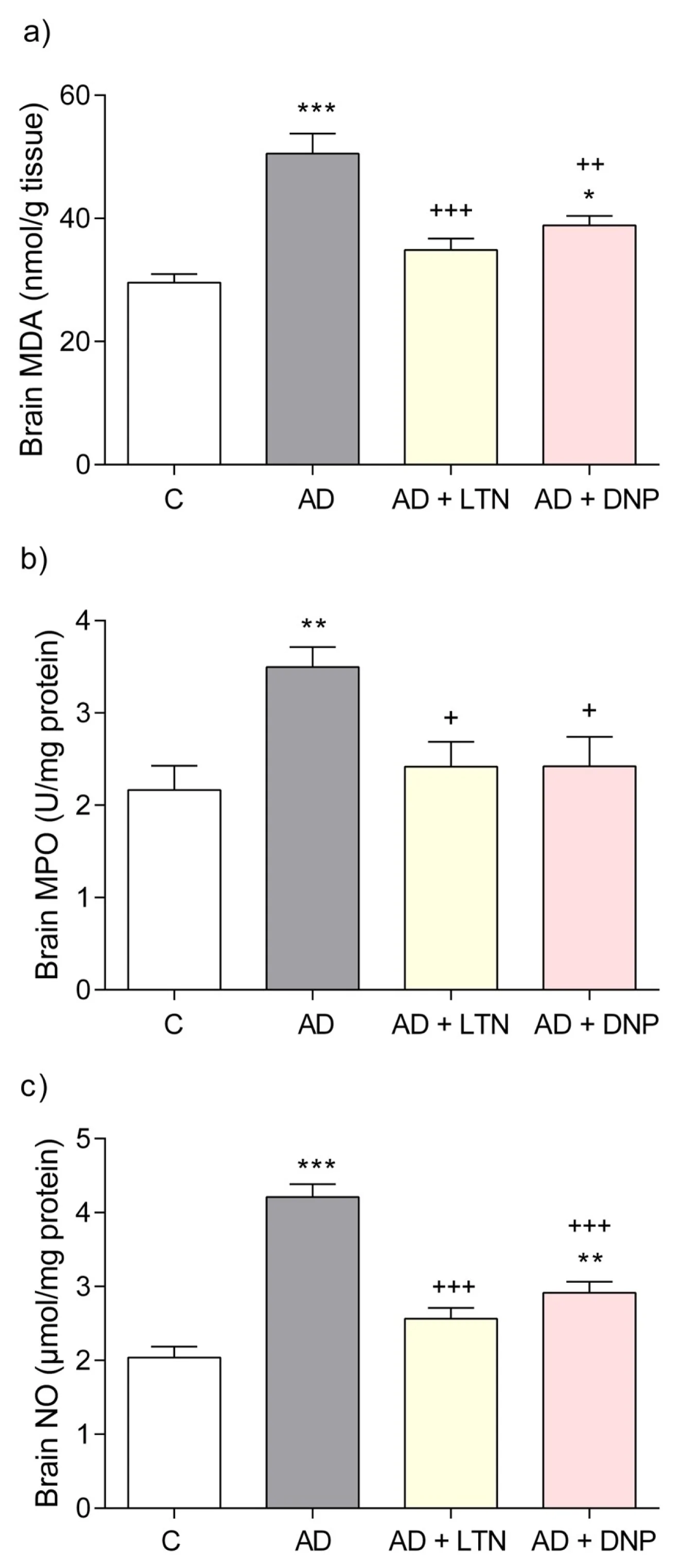
Effects of LTN on AlCl_3-_induced malondialdehyde (MDA) (a), myeloperoxidase (MPO) (b), and nitric oxide (NO) (c) levels in brain tissue. *p < 0.05, **p < 0.01, ***p < 0.001 versus control group; + p < 0.05, ++ p < 0.01, +++ p < 0.001 versus AD group. C: control; AD: Alzheimer’s disease; LTN: L-theanine; DNP: donepezil.

In the AD group, brain MDA levels (Figure 4a), an indicator of oxidative tissue damage, were significantly higher by 29% than those in the control group (p < 0.01), whereas they were significantly decreased in both treatment groups (AD + LTN and AD + DNP) by 31% and 24%, respectively (p < 0.001 and p < 0.01).

Brain MPO activity, which indicates neutrophil infiltration and inflammation, was significantly increased in the AlCl_3_-induced AD-like group (by 61%, p < 0.01), whereas LTN and DNP treatments significantly reduced MPO activity (by 31% and 30%, respectively; p < 0.05) (Figure 4b).

In the AD group, brain NO levels (Figure 4c), another indicator of oxidative tissue damage, were significantly higher by 104% than those in the control group (p < 0.01), whereas they were significantly decreased in both treatment groups (AD + LTN and AD + DNP) by 39% and 31%, respectively (p < 0.001).

**Figure 5:**
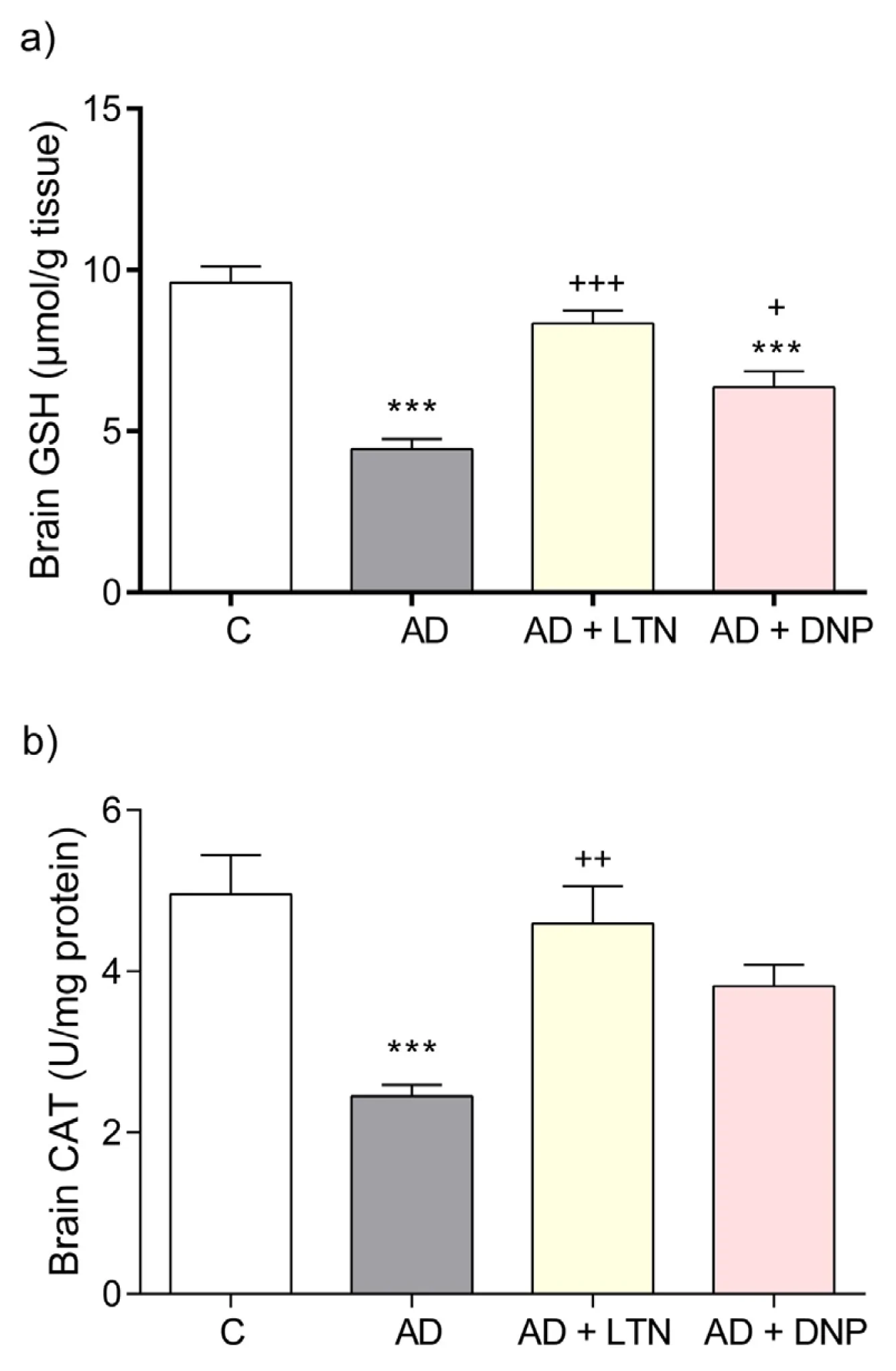
Effects of LTN on AlCl_3-_induced glutathione (GSH) levels (a) and catalase (CAT) activity (b) in brain tissue. ***p < 0.001 versus control group; + p < 0.05, ++ p < 0.01, +++ p < 0.001 versus AD group. C: control, AD: Alzheimer’s disease; LTN: L-theanine; DNP: donepezil.

As expected, due to increased oxidative stress in the AD group, tissue antioxidants, GSH and CAT, were significantly decreased compared with the control group (by 63% and 50%, respectively). In contrast, LTN treatment significantly restored both antioxidants (by 87% and 86%, respectively), whereas DNP treatment caused a significant increase only in GSH (by 43%). Although CAT levels did not reach statistical significance, they showed a tendency to increase (Figures 5a and 5b). Notably, DNP is not primarily an antioxidant agent. In contrast, the ability of LTN to increase GSH and CAT levels may be attributed to its antioxidant capacity, consistent with the literature [32].

## 4. Discussion

Aluminum, a neurotoxic agent thought to contribute to the etiology of AD, accumulates predominantly in the hippocampus and frontal cortex, which are critically important for cognitive function. Furthermore, studies have shown that aluminum interacts with proteins, leading to misfolding, aggregation, and hyperphosphorylation of proteins such as tau. Thus, oxidative stress has been demonstrated to contribute to the pathological mechanisms of AD by affecting processes such as Aβ accumulation, tau pathology, and mitochondrial dysfunction [33,34]. In the present study, the effects of LTN administration on neurotoxicity and cognitive impairment induced by AlCl_3_ exposure were investigated. In AD-like model rats, performance in both spatial and nonspatial memory tests was decreased, consistent with the neurotoxicity indicated by biochemical parameters.

AD is characterized by cortical and hippocampal degeneration, and short-term memory loss is among the earliest clinical manifestations. The novel object recognition test is widely used to evaluate memory [22,35]. In a previous study, animals administered AlCl_3_ showed significantly reduced performance in both the MWM test and NORT [36]. Similarly, in the present study, rats in the Alzheimer’s model group were found to exhibit significantly impaired performance in the NORT compared with the control group. However, memory performance was significantly improved following LTN treatment, as evidenced by increased time spent with the novel object and increased time in the target quadrant. Based on the results of cognitive function tests, AlCl_3_-induced memory impairments were found to be ameliorated by LTN. Specifically, oral administration of 100 mg/kg LTN has been shown to improve behavioral performance and restore antioxidant defense mechanisms (e.g., SOD, GSH, CAT) in experimental neurotoxicity models [19]. Moreover, dose-comparison studies have indicated that both 100 mg/kg and 200 mg/kg doses are effective, whereas increasing the dose provides only limited additional benefit, suggesting that 100 mg/kg represents an effective experimental dose [20].

Elbaz et al. [37] investigated the effects and underlying mechanisms of LTN on memory performance in mice with scopolamine-induced amnesia, a model commonly used in AD research. It was demonstrated that LTN improved oxidative parameters, suppressed inflammation, and reduced Aβ deposition, thereby supporting the findings of the present study.

In addition, the MWM test is commonly used to assess memory functions, particularly long-term and spatial memory, which are commonly impaired in AD [38–40]. In the present study, rats in the AlCl_3_-induced AD-like group were found to exhibit significantly impaired performance in the MWM. Following LTN treatment, the hidden platform was located more rapidly, and increased time was spent in the target quadrant during the probe phase of the test.

In both clinical and experimental models of AD, hyperphosphorylated tau and Aβ accumulation have been shown to contribute to neurodegeneration [41]. Increased levels of Aβ peptides have been shown to induce oxidative stress, leading to an elevation in reactive oxygen species (ROS) and subsequent neuronal loss [42]. Free radical molecules have been shown to attack lipids, proteins, and nucleic acids, thereby damaging their structures. The oxidative stress hypothesis has been proposed as a key mechanism underlying AD, given the high susceptibility of the brain and neural tissues to free radical-induced damage [43].

In the present study, AlCl_3_ administration significantly increased MDA levels, an indicator of lipid peroxidation in brain tissue. These findings align with previous research indicating that AlCl_3_-treated animals demonstrated a 40%–50% elevation in lipid peroxidation levels in brain tissue compared with baseline condition [44,45]. Similarly, El Rabey et al. [46] showed that MDA levels were significantly increased in the brain tissue of rats administered AlCl_3_, indicating the presence of oxidative damage.

MPO activity, another indicator of oxidative and inflammatory load, was also significantly elevated. Excessive MPO activity has been shown to enhance ROS generation and promote neuronal injury [47,48]. In the present study, MPO activity, examined as an indicator of oxidative damage and inflammation, was increased in brain tissue, whereas LTN treatment effectively suppressed MPO activity and prevented the elevation of tissue oxidants.

Moreover, in the present study, NO levels in the brain were found to be significantly increased in the AlCl_3_-induced AD-like neurotoxicity model. This increase may be considered an important indicator of neuroinflammation and oxidative stress. Although NO plays a role in synaptic plasticity and neurotransmission under physiological conditions, when produced at pathological levels, it has been shown to trigger the formation of ROS, such as peroxynitrite (ONOO-), and to cause neuronal damage and apoptosis [49,50]. The significant reduction in NO levels following LTN treatment may reflect its antioxidant and antiinflammatory properties [51].

Studies exploring the relationship between AD and oxidative damage have investigated various antioxidants against oxidative damage [46,52]. Elevated oxidative stress is known to impair endogenous antioxidant defenses; accordingly, GSH and CAT were decreased following aluminum administration in the present model. Based on the existing literature [19,53,54], which highlights the protective effects of LTN against oxidative tissue damage through its antioxidant and antiinflammatory properties, the potential neuroprotective effects of LTN against AlCl_3_-induced neurotoxicity in an experimental AD-like model were investigated. The findings of the present study demonstrated that LTN treatment markedly reduced brain MDA and NO levels, as well as MPO activity, which was accompanied by an increase in antioxidant enzyme levels.

El-Rabey et al. [46] similarly reported weakened antioxidant defenses in the brain tissue of AD-like rats induced by the AlCl_3_ model, along with decreased SOD and CAT levels and increased AChE activity and Aβ accumulation. In this study, a notable elevation in AChE activity and Aβ levels was found in parallel with increased oxidative stress. Gangarde et al. [38] examined cognitive function deterioration in AlCl_3_-induced AD-like model animals and emphasized that oxidative mechanisms are involved in the underlying damage and that ROS-mediated effects affect the cholinergic system.

In the literature, extensive evidence supports the antioxidant properties of polyphenolic compounds, catechins, and LTN, a bioactive component found in green tea. Indeed, it has been reported that these compounds improve age-related memory loss through their antioxidant properties [53], protect tissues in ischemia–reperfusion models [55], and provide neuroprotection in AD transgenic mice [56]. Some studies investigating the neuroprotective effects of LTN overlap with these antioxidant studies. Research conducted in animal models examining cognitive functions in relation to their neuroprotective effects has increased in recent years.

Zeng et al. [54] reported that LTN treatment in a D-galactose-induced AD-like model decreased advanced glycation end product and Aβ1–42 levels, while increasing ACh levels and inhibiting AChE activity. The same study also demonstrated that LTN suppressed the production of inflammatory factors and inflammation through the activation of sirtuin, a longevity-related gene, and strengthened the antioxidant defense system. The researchers suggested that these effects may improve quality of life, particularly in patients with AD.

Park et al. [57] reported that LTN treatment following trauma in a traumatic brain injury (TBI) model effectively reduced neuronal death and cognitive impairment by suppressing α-amino-3-hydroxy-5-methyl-4-isoxazolepropionic acid receptor activation and neuroinflammation. Significant improvements in cognitive function were also demonstrated in the probe test of the MWM.

The findings of the present study are consistent with the cholinergic hypothesis, which suggests that increased AChE activity in the brain, such as that induced by aluminum exposure, contributes to AD pathology. Additionally, mechanisms triggered by oxidative damage are consistent with AD pathogenesis through amyloid accumulation in tissues. Roy et al. [58] reported that, based on their analysis of the relationship between oxidative stress and Aβ accumulation/tau hyperphosphorylation, oxidative stress acts as an initial factor in the pathogenic process. The marked increase in AChE activity observed in the AlCl_3_ group may be related to the relatively severe cholinergic and oxidative disruption induced by cumulative aluminum exposure in the present dose-escalation protocol. Likewise, the pronounced improvement observed following LTN treatment may be associated with its antioxidant and neuroprotective properties. Nevertheless, this degree of biochemical recovery should be interpreted in the context of the severity of the experimental model and its specific biochemical characteristics.

One limitation of this study is the lack of histopathological evaluation to confirm amyloid plaque deposition and tau-related pathology. While the biochemical findings support Alzheimer-like neurotoxicity, direct morphological evidence of plaque formation or tau hyperphosphorylation was not assessed. In addition, DNP was administered intraperitoneally, whereas LTN and AlCl_3_ were administered orally; therefore, route-related pharmacokinetic differences should be considered when interpreting the treatment effects.

In conclusion, considering its previously reported neuroprotective properties, the potential of LTN, a natural bioactive amino acid found in tea leaves, as an alternative therapeutic strategy in an AD-like model was investigated. The present study demonstrated that 4-week AlCl_3_ exposure induced oxidative stress and reduced cholinergic activity, leading to cognitive impairment. To the best of our knowledge, this is the first study to evaluate the oxidative, cholinergic, and cognitive effects of LTN in an AlCl_3_-induced AD-like model. LTN administration markedly improved these behavioral and biochemical parameters, exhibiting therapeutic efficacy comparable to that of DNP. Unlike conventional AChE inhibitors, LTN is a naturally occurring bioactive molecule, and the findings of the present study suggest that it may have potential as a functional food supplement for the prevention of AD and the promotion of healthy aging.
